# Developmental changes of cortical white–gray contrast as predictors of autism diagnosis and severity

**DOI:** 10.1038/s41398-018-0296-2

**Published:** 2018-11-16

**Authors:** Gleb Bezgin, John D. Lewis, Alan C. Evans

**Affiliations:** Montreal Neurological Institute, McGill University, Montreal, Canada

## Abstract

Recent studies suggest that both cortical gray and white-matter microstructural characteristics are distinct for subjects with autism. There is a lack of evidence regarding how these characteristics change in a developmental context. We analysed a longitudinal/cross-sectional dataset of 402 magnetic resonance imaging (MRI) scans (171 subjects with autism and 231 with typical development) from the Autism Brain Imaging Data Exchange, cohorts I–II (ABIDE-I-II). In the longitudinal sample, we computed the rate of change in the white–gray contrast, a measure which has been related to age and cognitive performance, at the boundary of the cerebral cortex. Then, we devised an analogous metric for the cross-sectional sample of the ABIDE dataset to measure age-related differences in cortical contrast. Further, we developed a probabilistic model to predict the diagnostic group in the longitudinal sample of the cortical contrast change data, using results obtained from the cross-sectional sample. In both subsets, we observed a similar overall pattern of greater decrease within the autistic population in intensity contrast for most cortical regions (81%), with occasional increases, mostly in primary sensory regions. This pattern correlated well with raw and calibrated behavioural scores. The prediction results show 76% accuracy for the whole-cortex diagnostic prediction and 86% accuracy in prediction using the motor system alone. Our results support a contrast change analysis strategy that appears sensitive in predicting diagnostic outcome and symptom severity in autism spectrum disorder, and is readily extensible to other MRI-based studies of neurodevelopmental cohorts.

## Introduction

Autism spectrum disorder (ASD) is a complex and heterogeneous cluster of developmental abnormalities characterised by disrupted social reciprocity, repetitive behaviours and restricted interests^1^. Such behavioural abnormalities are found to have certain brain-structural and physiological correlates on the level of the cortical gray matter (GM)^2–4^. It has also been found that ASD features not only GM aberrations, but also abnormal white-matter (WM) microstructure^5–7^, specifically evident as age-related difference between ASD and typical development (TD) in adolescent developmental trajectories^8^. Combining cortical features of GM and WM in one analysis might provide a more complete understanding of the disorder. One way to establish such a combination is to compute the ratio of MRI intensities sampled in- and outside of the white–gray matter boundary, referred to as white–gray contrast (WGC). Such a contrast metric, proposed almost a decade ago^9^, reflects both gray and white-matter properties and has proven sensitive in predicting such features as biological age and cognitive performance with high accuracy^10,11^. Utilisation of this metric in the autism domain is relatively new. A histological study^12^ investigated the white–gray matter boundaries in post-mortem tissue using sigmoid curves to quantify cell distributions in the white–gray transition zones, and found that the curves were steeper for TD subjects than for individuals with ASD, suggesting poorer contrast for the latter. In a recent MRI study^13^, the authors sampled intensity across multiple distances from the white–gray matter boundary. The results consistently yielded a greater decrease in ASD of a measure analogous to WGC, particularly in bilateral temporal regions. In this study, we take this approach one step further, taking into account the fact that ASD is a developmental disorder and thus crucial information can be retrieved from age-related neuroanatomical changes^14,15^. Hence, instead of measuring absolute ASD-TD group differences in WGC, we assess how this contrast changes with age, in longitudinal and cross-sectional contexts.

An additional strength of the WGC is that cortical contrast measures seem generally less affected by erroneous brain registration and inherent irregularities in tissue intensities^16^. Such problems, in combination with poor signal-to-noise ratio in MRI images resulting from insufficient or absent quality control, heterogeneities related to symptoms, gender, age and data collection sites often render observations unstable—occasionally providing even null results^17^—that contribute to the confusing state of the literature on neuroanatomical and neurophysiological correlates of ASD^18–22^.

To increase sample sizes and gain a better understanding of the structural phenotype for autism, several ASD data agglomeration initiatives have recently emerged^23–26^. One such initiative, the Autism Brain Imaging Data Exchange^23,24^ (ABIDE), assembled brain imaging and behavioural phenotypic information from 25 international sites.

We analysed a large sample of MRI data from the ABIDE database, featuring predominantly the age span of adolescence, including both cross-sectional and longitudinal samples, using multivariate techniques, and compared the age-related change of white–gray contrast (WGC) to behavioural phenotypic measures. We hypothesised that age-related WGC changes would be different between ASD and TD groups, and that these differences would be similar in longitudinal and cross-sectional samples. To further evaluate this hypothesis, we developed a novel probabilistic approach for predicting the diagnostic outcome given longitudinal cortical contrast change from cross-sectional data.

## Materials and methods

### ABIDE-I and ABIDE-II databases

The Autism Brain Imaging Data Exchange (ABIDE) database emerged as a large-scale multi-site initiative to assemble structural, functional and diffusion magnetic resonance imaging (MRI) data, along with accompanying phenotypic descriptions^23^. Recently, this dataset has been substantially augmented by the inclusion of a more extensive sample, including longitudinal data, referred to as ABIDE-II^24^. For most ASD subjects in the sample, a comprehensive phenotypic inventory is provided, including various scores relevant for symptom evaluation. In the current study, we use the structural images, and the Autism Diagnostic Observation Schedule (ADOS) scores^27^.

### MRI data processing

#### Surface extraction

The T1-weighted MRI data constituting 1031 images from ABIDE-I and 1263 images from ABIDE-II were processed with CIVET^28^ (version 2.1, released November 2016), a fully automated structural image analysis pipeline developed at the Montreal Neurological Institute. CIVET corrects intensity non-uniformities using non-parametric non-uniform intensity normalisation^16^ (N3), aligns the input volumes to the Talairach-like ICBM-152-nl template^29^, classifies the image into white matter, gray matter, cerebrospinal fluid, and background^30,31^, extracts the white-matter and pial surfaces^28^, and maps these to a common surface template^32^. These results were then subjected to a manual quality control to ensure that the white-matter surface was correctly placed at the inner edge of the cortical gray matter. The placement of the pial surface was ignored; the measures of white/gray contrast are based only on the white surface. White/gray contrast was measured on the data that passed this quality control.

#### White–gray contrast (WGC)

White–gray contrast was measured as the ratio of the intensity on the T1-weighted MRI 1 mm inside the white surface to the intensity 1 mm outside the white surface (Supplementary Figure 1; see also Fig. 1 in ref. 10). To obtain these measures, a distance map was created from the white surface at 0.25 mm resolution; the distance map was smoothed with a 0.5 mm full width at half maximum (FWHM) Gaussian kernel; and a gradient vector field was computed. A copy of the white surface was then moved 1 mm inward along this gradient vector field to produce a sub-white surface, and a copy was moved 1 mm outward to produce a supra-white surface. The T1-weighted intensity values were sampled at each vertex of both the supra-white surface and the sub-white surface. These values were smoothed with a 20 mm FWHM Gaussian kernel on the MNI152 average surface, and then the ratio was computed by dividing the value at each vertex of the sub-white surface by the value at the corresponding vertex of the supra-white surface.

**Fig. 1:**
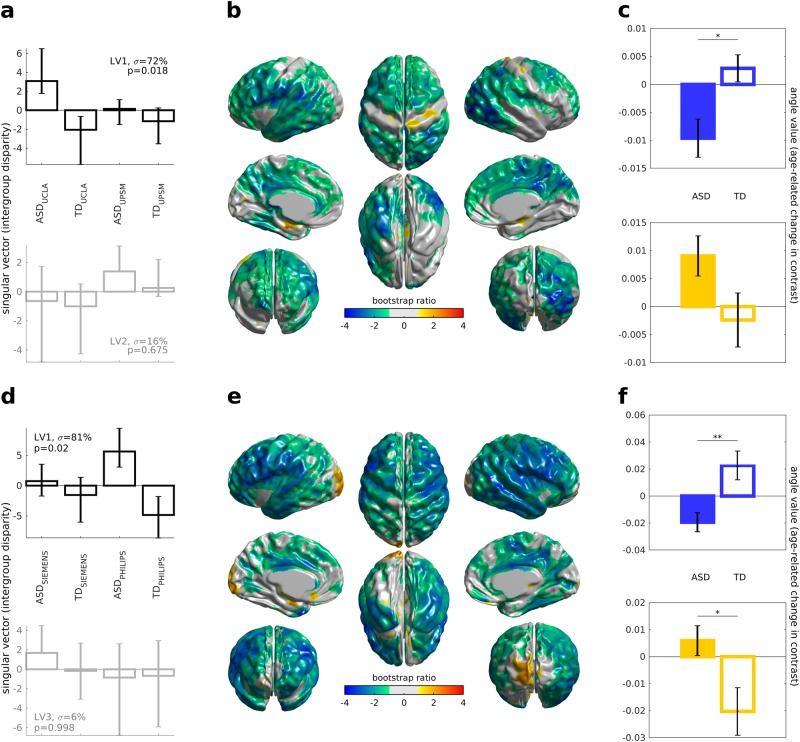
PLS analysis of the longitudinal and cross-sectional samples of the ABIDE dataset. **a**–**c** Longitudinal sample PLS results; **d**–**f** Cross-sectional sample PLS results. **a** Top: group-related singular vector from the first latent variable (LV1) captures inter-group differences related to diagnosis rather than to site; error bars depict confidence intervals from the bootstrap samples. **a** Bottom: a pattern most similar to between-site difference is captured by LV2 and appears non-significant. **b** Bootstrap ratio (BSR) values depicting the cortical pattern captured by LV1. **c** Top: change of MRI contrast in the vertices within the range between the lowest BSR and one standard deviation above that value. **c** Bottom: change of MRI contrast in the vertices within the range between one standard deviation below the highest BSR and the highest BSR. **d**, **e** Analogous depiction of PLS results for the cross-sectional sample; **d** Bottom: a pattern most similar to between-site difference is captured by LV3 and appears non-significant. **f** Contrast change distributions for the cross-sectional sample, analogous to those in **c** for the longitudinal sample

#### Resulting samples

After the quality control and application of all the exclusion criteria, listed below, the resulting samples included 380 subjects (158 ASD). Namely, the CIVET procedure failed to process 186 images, 612 images survived the quality control, of which there were 115 female scans and 73 images from 17 sites falling into groups containing less than four subjects, and were removed. To ensure independence in comparisons and predictions, we have separated the resulting dataset into two non-overlapping samples: cross-sectional denoted *crsc*, and longitudinal denoted *lngt*.

#### Cross-sectional sample

In the cross-sectional sample, sites containing less than four subjects in either group were excluded from the analysis; only male subjects were included in both diagnostic groups, ASD and TD. The site ABIDEII-SDSU_1 was excluded from the analysis as the only site that utilised a scanner from a manufacturer other than Siemens or Philips, with a negligible contribution to the sample. The part of the sample that represented longitudinal data was excluded to ensure cross-sectional and longitudinal samples do not overlap (see the full lists of these samples in Supplementary Text 2). The resulting cross-sectional sample consisted of 359 subjects (146 ASD; see Table 1).

**Table 1:** Cross-sectional sample of ABIDE data that passed quality control

Acquisition site	nr. ASD’s	nr. TD’s	nr. subjects	Age, ASD	Age, TD	Scanner
CMU	6	6	12	22.5 ± 2.6	24.7 ± 4.8	Siemens Magnetom Verio syngo MR B17
KKI	11	17	28	10.4 ± 1.5	10.2 ± 1.2	Philips Achieva
LEUVEN_1	8	10	18	22.4 ± 3.2	24.0 ± 3.0	Philips Intera
LEUVEN_2	8	8	16	13.4 ± 1.1	14.9 ± 1.5	Philips Intera
NYU	25	30	55	14.5 ± 7.0	12.3 ± 3.9	Siemens Magnetom Allegra syngo MR 2004A
TRINITY	13	11	24	16.6 ± 2.9	17.1 ± 3.7	Philips Achieva
UCLA_1	6	9	15	14 ± 3.6	13.4 ± 1.5	Siemens Magnetom TrioTim syngo MR B15
ABIDEII-ETH_1	6	17	23	21.3 ± 4.4	22.6 ± 4.5	Philips Achieva
ABIDEII-GU_1	9	15	24	10.7 ± 1.7	10.7 ± 1.5	Siemens Magnetom TrioTim syngo MR B17
ABIDEII-KKI_1	10	44	54	11.0 ± 1.3	10.5 ± 1.2	Philips Achieva
ABIDEII-NYU_1	22	16	38	8.8 ± 3.0	8.8 ± 1.8	Siemens Magnetom Allegra syngo MR 2004A
ABIDEII-OHSU_1	6	7	13	12.0 ± 2.7	8.9 ± 0.7	Siemens Magnetom TrioTim syngo MR B17
ABIDEII-TCD_1	12	18	30	14.3 ± 3.3	15.7 ± 3.2	Philips Intera Achieva
ABIDEII-UCLA_1	4	5	9	12.0 ± 1.8	10.8 ± 2.9	Siemens Magnetom TrioTim syngo MR B17
Total	146	213	359	13.6 ± 5.4	13.7 ± 5.4	Siemens (168 subjects); Philips (193 subjects)

Age shown in format ‘mean ± s.d.’

#### Longitudinal sample

The resulting analysis on the longitudinal sample included 42 images from UCLA and UPSM data collection sites (12 subjects with ASD and 9 with TD, times two time points; mean age at baseline scan 12.75 y.o., mean age at the follow-up scan 15.06 y.o.; 3 females (1 ASD); see Supplementary Table 1).

#### Proxy calibrated severity scores

Among the phenotypic information in the ABIDE data are the Autism Diagnostic Observation Schedule^23^ (ADOS) scores for each ASD subject. ADOS represents a semi-structured assessment of social interaction, communication and stereotypical behaviours for individuals with ASD. The ADOS applies to children as well as adults, and to individuals ranging from non-verbal to verbally fluent. But, different ADOS modules are utilized for individuals at different stages of development, or different language abilities, and the scores from different modules are not directly comparable. To allow comparison of ADOS scores across modules, these raw ADOS scores can be transformed into calibrated severity scores^33,34^. These calibrated severity scores utilize specific subsets of items within each module to create a more comparable algorithmic score^33^, followed by an additional adjustment for age and ADOS module^34^. The ABIDE data do not provide all of the information necessary to faithfully apply this correction, but a proxy severity score can be derived using the total of the social and communication ADOS scores in place of the algorithm score^35^. In this work, we relate contrast change to these proxy severity scores, as well as to the total of the social and communication ADOS scores.

### Statistical analysis

In the longitudinal sample, to account for age-related differences in WGCs, we devised a metric, called “angle”, capturing how fast WGC changes with age, computed for each vertex and denoted *α*, wherein the rate of change in the WGC had a linear dependency on the difference between age at baseline scan and age at follow-up scan:

1 $$\alpha _i^{lngt} = \arctan \left( {\frac{{{\rm WGC}_i^{flp} - {\rm WGC}_i^{bsl}}}{{{\rm age}_i^{flp} - {\rm age}_i^{bsl}}}} \right),$$

where $\alpha _i^{lngt}$ is an angle of subject *i*, proportional to the difference between WGC of the follow-up and WGC of the baseline (denoted ${\rm WGC}_i^{flp}$ and ${\rm WGC}_i^{bsl}$, respectively). As this difference represents the opposite cathetus for the given angle and the age difference between follow-up and baseline visits is the adjacent cathetus, the resulting angle is computed from the opposite-adjacent division using the arctangent function.

As an analogous angle metric for the cross-sectional sample, we have proposed the following measure, computed as a mean pairwise WGC change with age, for each subject and the rest of the subjects from the same diagnostic group within a data collection site:

2 $$\alpha _i^{crsc} = \arctan \left( {\frac{{\mathop {\sum }\nolimits_{j = 1:N_s^{dx}}^{j \ne i} \left( {{\rm WGC}_i - {\rm WGC}_j} \right)}}{{\mathop {\sum }\nolimits_{j = 1:N_s^{dx}}^{j \ne i} \left( {{\rm age}_i - {\rm age}_j} \right)}}} \right),$$

where $\alpha _i^{crsc}$ is computed for each vertex by obtaining the average difference between the WGC of the *i*th subject (denoted WGC_*i*_) and the WGCs of all the other $N_s^{dx}$ subjects from the same diagnostic group *dx* (either ASD or TD) within a given acquisition site *s*, divided by the corresponding average age difference between subject *i* and the ages of the rest of subjects from that site, and taking the arctangent from the resulting fraction. This definition essentially represents the per-subject specification of a developmental trajectory and provides a benefit of diminishing inherent site effects. All the subsequent analyses in this study will use this definition as a metric, rather than a single time point WGC estimate.

The metrics *α*^*lngt*^ and *α*^*crsc*^ were subjected to partial least squares (PLS) analysis^36^, which was designed to identify common brain patterns for a given set of diagnostic groups or experimental conditions. PLS makes use of singular value decomposition (SVD) to re-express the data as latent variables (LV), akin to eigenvectors in principal component analysis. For that analysis, the *α*^*lngt*^ values were split into four groups: ASD_UCLA_, TD_UCLA_, ASD_UPSM_ and TD_UPSM_, corresponding to individuals diagnosed with autism and scanned at the UCLA site, those featuring typical development and scanned at the UCLA site, subjects diagnosed with autism and scanned at the UPSM site and those featuring typical development and scanned at the UPSM site, respectively. To ensure the same number of LVs in the PLS analysis of the cross-sectional sample, we split the *α*^*crsc*^ values into the following four groups, arranging the angle data by diagnostic groups and scanners: ASD_SIEMENS_, TD_SIEMENS_, ASD_PHILIPS_ and TD_PHILIPS_. Following SVD, PLS analysis performs two instances of statistical testing, permutation and bootstrapping. The former technique assesses statistical significance by resampling without replacement to reassign the order of groups for each subject. For each new sample, PLS is recalculated, counting the number of times the permuted singular values exceed the original calculation output, resulting in a null hypothesis probability. Conversely, bootstrapping assesses the reliability by resampling with replacement while keeping the group assignment fixed, providing confidence intervals for each group. To incorporate this reliability in the singular vector representing cortical patterns, a set of bootstrap ratio values (BSRs) is obtained by means of dividing the cortical representation of the group differences by the bootstrap standard errors. To assess how strongly each subject expresses the pattern on a given LV, the metric called brain score (abbreviated as BrSc) is obtained by means of multiplying the original mean-centred matrix with the angle values by the matrix with BSR values.

#### Predictive models for longitudinal data using cross-sectional samples

The model to predict diagnostic group from angle values was devised in a Bayesian setting:

3 $$P\left( {dx{\mathrm{|}}\alpha } \right) \propto P\left( {\alpha {\mathrm{|}}dx} \right) \times P\left( {dx} \right),$$

where *P*(*dx*|*α*) is a posterior probability of a diagnosis given the angle wherein the angle values were standardised to emphasise shape of a distribution over its magnitude, *P*(*α*|*dx*) is a likelihood of angle value given the diagnosis, and *P*(*dx*) is the prior probability representing acquired knowledge about the diagnosis. The likelihood term was represented by an extreme value distribution of the form:

4 $$P\left( {\alpha {\mathrm{|}}dx} \right) = \sigma ^{ - 1}\exp \left( {\frac{{ \pm \alpha - \mu }}{\sigma }} \right) \times \exp \left[ { - \exp \left( {\frac{{ \pm \alpha - \mu }}{\sigma }} \right)} \right],$$

where *σ* is the scale parameter of the distribution, *α* is the set of cross-sectional angle values and *μ* is the location parameter of the distribution. This distribution was represented in a generalised form wherein the skewness direction was regulated by the sign of *α* dependent on the best fit to the actual *α* values, measured using Pearson correlation. The prior parameter was informed by bootstrap ratio (BSR) values derived using PLS analysis and represented by a sigmoid function of the form:

5 $$P\left( {dx} \right) = \left[ {1 + \exp \left( { \pm \frac{{x \times {\rm BSR}}}{{{\rm BSR}_{{\rm max}}}}} \right)} \right]^{ - 1},$$

where $x \in \left[ {\alpha _{{\rm min}}^{crsc},\alpha _{{\rm max}}^{crsc}} \right]$ and the sign in the round brackets is positive if *dx* = *TD* (monotonically increasing if BSR < 0) and negative if *dx* = ASD (monotonically increasing if BSR > 0; see Results for details on such prior choice). To emphasize the shape of distributions over their magnitude, both *α*^*lngt*^ and *α*^*crsc*^ were *z*-scored. This model is summarised in Supplementary Figure 2.

A second model was devised to predict proxy severity scores^35^ in the longitudinal subset from *BrSc* values in the cross-sectional subset:

6 $$S_i^{lngt} = \beta _0^{crsc} + \beta _1^{crsc} \ast BrSc_i^{crsc} + \frac{{\mathop {\sum }\nolimits_{i - w < k < i + w} \varepsilon _k}}{K},$$

where $S_i^{lngt}$ is a predicted proxy calibrated severity score for the *i*th subject in the longitudinal subset, $\beta _0^{crsc}$ is an intercept in the severity general linear model (GLM) constructed from the cross-sectional sample (with *BrSc* being the independent variable), $\beta _1^{crsc}$ is that model’s slope, and the rightmost term is representing mean residual error from the window surrounding the *i*th *BrSc* value with an interval *w* set to be equal to 5% from the maximum *BrSc* value; considering there are *K* data points within such an interval, *ε*_*k*_ represents the residual error for each of them. Of note, ABIDE-II provides a single-score ADOS information for each subject in the longitudinal sample, i.e. there is no information on the per-subject ADOS difference between baseline and follow-up visits.

### Code availability

CIVET pipeline: http://www.bic.mni.mcgill.ca/ServicesSoftware/CIVET-2-1-0-Table-of-Contents

## Results

### PLS analysis results

The two instances of PLS analyses yielded similar cortical distributions (Fig. 1), with an overall greater cortical contrast decrease for ASD as compared to TD across the cortical mantle, but with occasional focal increases (in early visual, medial temporal and somatomotor areas) in ASD as compared to TD.

### Longitudinal PLS results

For the four groups in the longitudinal sample (ASD_UCLA_, TD_UCLA_, ASD_UPSM_ and TD_UPSM_), the first latent variable (LV) (*p* = 0.018, permutation test) from the PLS analysis yielded the following design scores: (0.79, −0.53, 0.04, −0.3). The alternating sign of this first LV indicates that the most covariance in the data (72%) was explained by diagnostic groups rather than sites (Fig. 1a, top). The between-group difference patterns normalized by a standard error across the bootstraps (bootstrap ratio, BSR; Fig. 1b) showed a distribution suggesting slightly greater increase of white–gray contrast (WGC) patterns within early visual areas, bilateral sensorimotor strip, and a scattered set of ventral stream regions among the ASD subjects. Conversely, a greater increase (*p* < 0.05, two-sample *t*-test for the vertices within one standard deviation from the minimum BSR) of WGC among the TD group was observed in a wide network of regions spanning large portions of lateral and medial frontal, parietal and posterotemporal areas in particular (Fig. 1c, top).

### Cross-sectional PLS results

The four groups assembled for the PLS analyses for the cross-sectional data (ASD_SIEMENS_, TD_SIEMENS_, ASD_PHILIPS_ and TD_PHILIPS_) showed a similar pattern of overall greater decrease of the cortical tissue contrast for the ASD subjects as compared to TD controls (design scores: (0.11, −0.2, 0.74, −0.64); see Fig. 1d). The most prominent WGC increase in ASD as compared to TD was observed in early visual areas (particularly in the left hemisphere), most of the remaining cortex featured predominant decrease in ASD as compared to TD (Fig. 1e, f).

**Fig. 2:**
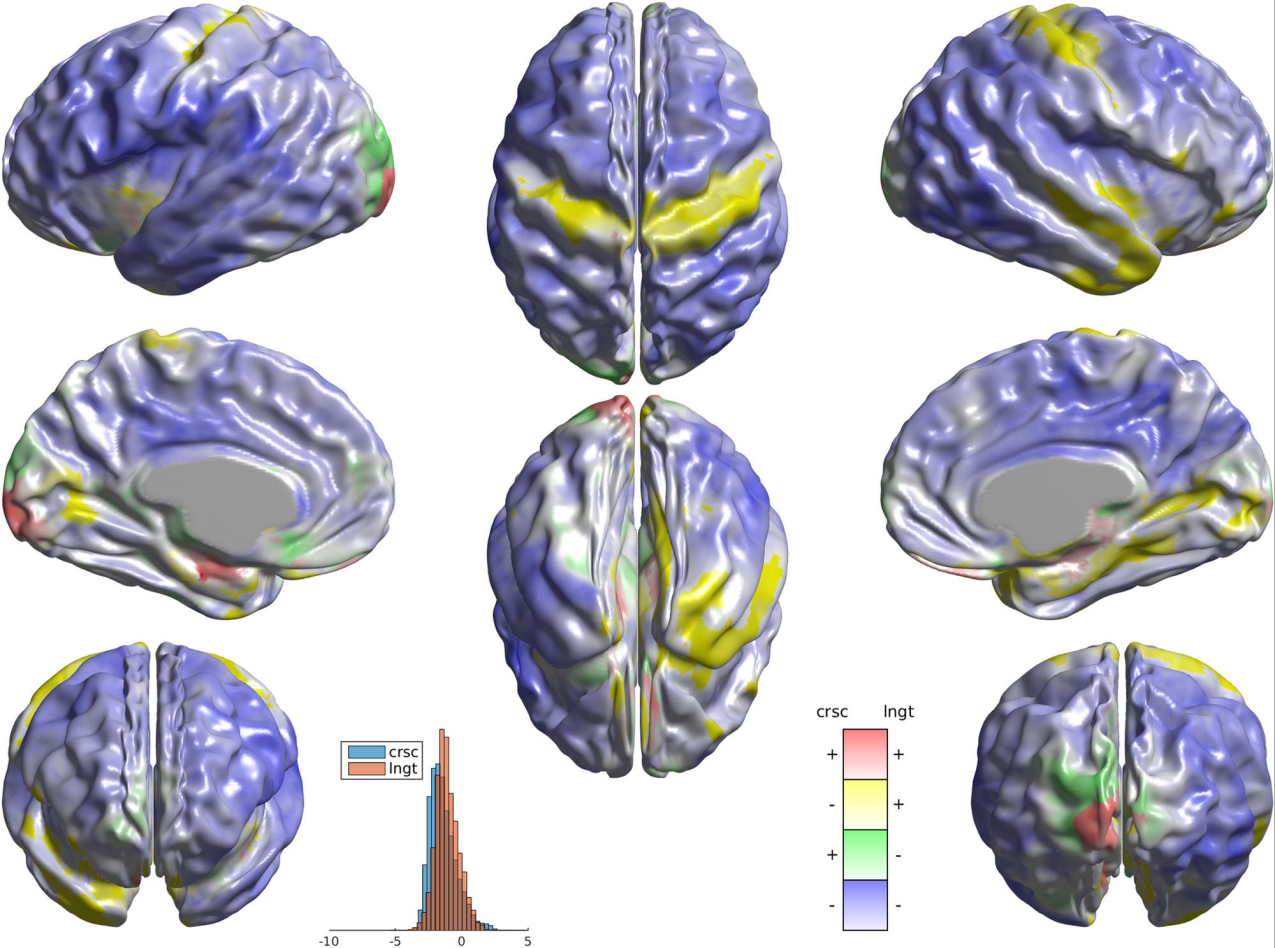
Conjunction analysis of PLS outputs for longitudinal and cross-sectional samples. Blue colours, covering 81% of the cortex, indicate negative bootstrap ratio (BSR) values in both samples, shades of red stand for mutually positive BSRs. Green shades indicate positive BSR in the cross-sectional and negative BSR in the longitudinal samples, while yellow colours stand for longitudinal positive and cross-sectional negative. The histograms depict BSR value distributions within longitudinal and cross-sectional samples. Colour intensities reflect BSR value magnitude, averaged across longitudinal and cross-sectional samples

Both longitudinal and cross-sectional instances of PLS analysis showed a broad set of regions across the cortex featuring greater decrease in WGC with age in ASD compared to typically developing subjects; indeed, vertices with a mutual decrease in both samples represent 81% of the entire cortical sheet (Fig. 2). Notably, considering WGC as a static measure per subject (no age-related change), an analogous PLS analysis yielded no result: the latent variable capturing most of diagnostic difference explained 0.27% of covariance (*p* = 0.74, permutation test; result not shown).

**Fig. 3:**
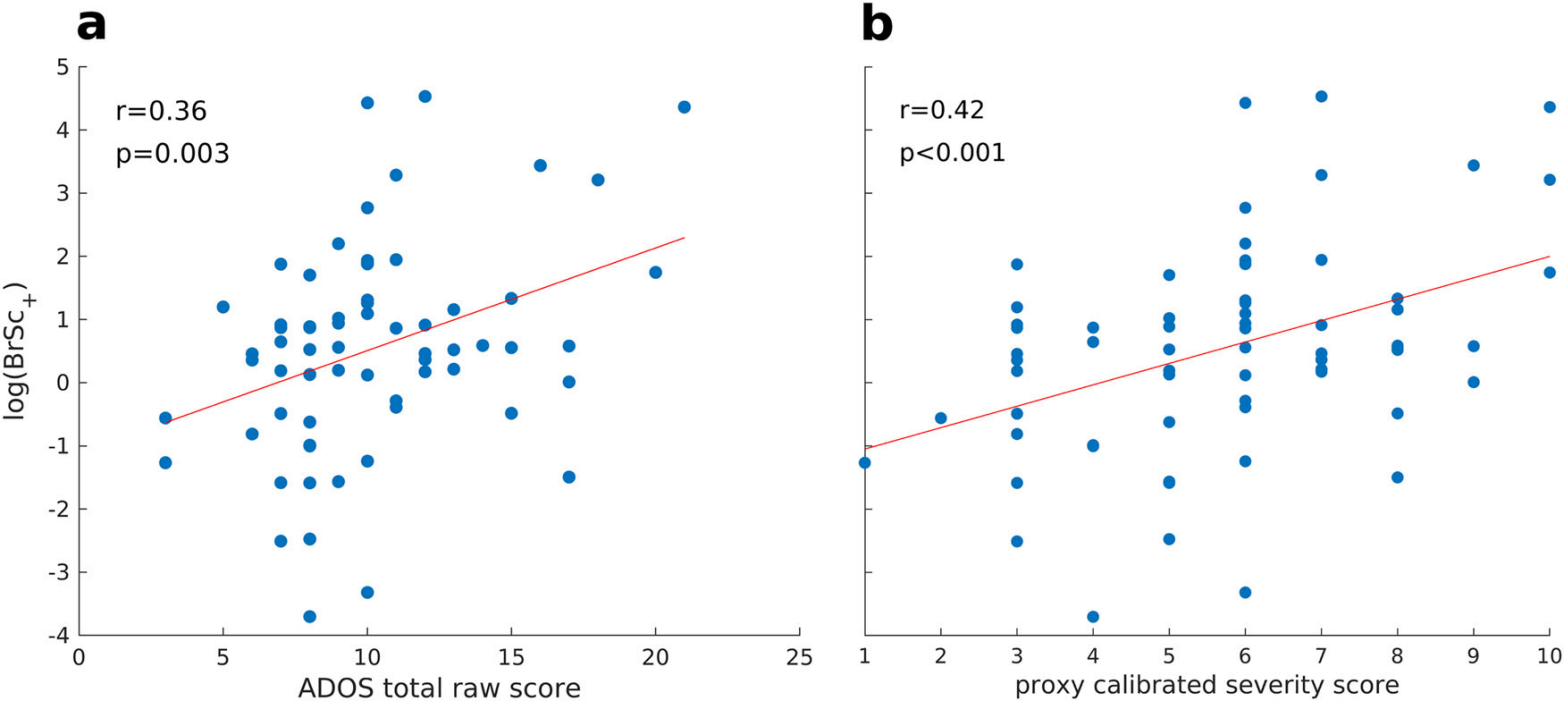
Correlations between phenotypic scores and PLS-derived brain scores in the cross-sectional sample of the ABIDE database. **a** Correlation between raw total ADOS scores and natural logarithm of positive brain scores. **b** Correlation between proxy calibrated severity scores and natural logarithm of positive brain scores

### Relation to behavioural metrics

We observed correlation between raw ADOS scores and the positive part of *BrSc* values on the log scale, denoted log(*BrSc*_+_), suggesting that the more ASD subjects expressed the pattern on the LV1, capturing the most difference between the two diagnostic groups, the more severe the symptoms were (*r* = 0.36, *p* < 0.005; Fig. 3a). A greater correlation was observed between ADOS proxy calibrated severity scores and log(*BrSc*_+_) (*r* = 0.42, *p* < 0.001; Fig. 3b).

### Longitudinal prediction results

We devised a model to predict diagnostic assignment in the longitudinal sample using the training data from the cross-sectional sample (Supplementary Figure 2). The angle value histograms are well approximated with extreme value distributions that use location parameter *μ* and scale parameter *σ* (Supplementary Figure 2C). Information from PLS analysis can be used to inform prior distributions: if the BSR value is negative, indicating a contrast decrease for ASD as compared to TD, the simplest way to create a prior distribution is to form a sigmoid curve with a negative slope for the ASD sample and a positive slope for the TD sample, with its steepness informed by the given BSR value (Supplementary Figure 2D). The opposite holds if the BSR value is positive. Ultimately, using likelihood and prior functions, the posterior probability obtained is used to predict a diagnostic outcome from a given angle value (Supplementary Figure 2B).

**Fig. 4:**
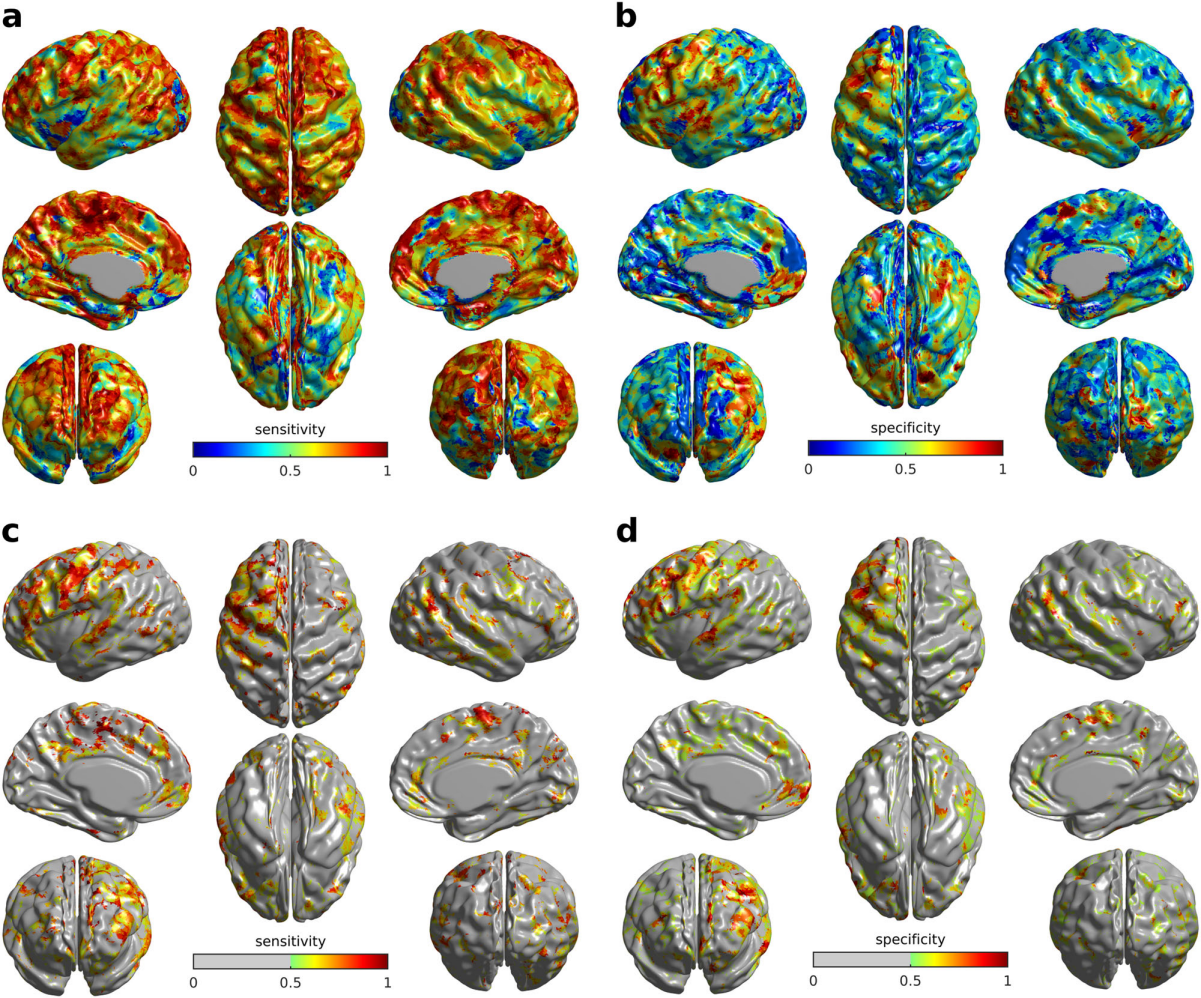
Sensitivity and specificity of cross-sectional/longitudinal predictions from individual vertex models. **a** Cortical model sensitivity distribution. **b** Cortical model specificity distribution. **c**, **d** Sensitivity and specificity maps thresholded at a condition of both being higher than 50%

We will refer to the fraction of correctly predicted ASD diagnosis outcomes as sensitivity, and the fraction of correctly predicted TD outcome as specificity. Using the mean cortical distribution of angle values, our model resulted in 83% sensitivity (10 out of 12 correctly predicted ASD outcomes) and 67% specificity (6 out of 9 correctly predicted TD outcomes; see Supplementary Table 1, last column), thus achieving overall accuracy of 76%. Notably, prediction based on bilateral motor regions alone yielded 89% specificity while retaining the whole-cortex model sensitivity, resulting in 86% overall accuracy. Using single-vertex models at each of the vertices in the average template, we investigated cortical distributions of sensitivity and specificity (Fig. 4), including the 50% mutual cutoff representation (Fig. 4c, d) to avoid trivial cases such as 100% sensitivity and 0% specificity and vice versa. Analysis of cortical vertices that contributed to both >50% sensitivity and >50% specificity suggested a generally left-lateralised pattern confined predominantly to the lower sensorimotor strip and temporo-parietal junction (Fig. 4c, d; see Supplementary Figure 3 for the sensitivity-specificity conjunction result).

### Prediction of behavioural scores

The proposed model for predicting severity scores in the longitudinal sample using BrSc values in the cross-sectional sample (Eq. 6) resulted in the following outcomes. The correlation between actual proxy calibrated severity scores in the longitudinal sample and their counterparts predicted from the cross-sectional sample was marginally significant (*r* = 0.54, *p* = 0.084). The correlation between the longitudinal sample proxy calibrated severity scores (independent variable) and raw ADOS scores, predicted from the BrSc value in the cross-sectional sample (dependent variable), was strong and significant (*r* = 0.63; *p* < 0.05).

## Discussion

The finding of overall greater contrast decrease in ASD than in TD is consistent with previous studies^12,13^, while extending their original notions with age-related contrast change. Vertices exhibiting greater contrast decrease in TD than in ASD displayed a regional distribution weighted toward primary sensory areas; this distribution was similar for both longitudinal and cross-sectional samples. The main difference constituted a greater somatosensory increase in the longitudinal sample for ASD subjects (whereas cross-sectional data indicated an increase only in a small dorsal portion of the left somatosensory cortex, see Fig. 2), and a greater primary visual increase for ASD subjects in the cross-sectional sample (with a similar but less prominent pattern in the longitudinal sample). These primary sensory patterns are likely reflecting complex cellular processes: whereas the rates of overall axonal pruning and other related processes such as apoptosis and cell migration may be reduced in autism^6^, resulting in lower contrast, dendritic arborisations increase dramatically in the primary sensory areas during the normal development^37^, resulting in an increased gray matter intensity and hence reduced WGC in those regions, while the opposite generally holds for secondary and association areas^10^. Such increased dendritic arborisations in middle cortical layers normally appear early in development, during the formation of the cortical subplate, as thalamocortical afferents reach the primary cortical regions^38^. This process affects primary visual, auditory and somatosensory regions to a similar extent and nearly simultaneously, resulting in an increased myelination thereof^39^, affecting physiology and corresponding perceptions^40^. This process might be disrupted in ASD and manifest itself as an increased contrast compared to TD. Consistent with this notion, a recent MRI study^41^ observed greater gray matter volume and resting state functional connectivity in the somatosensory cortex of children diagnosed with ASD than in that of their TD peers. Conversely, most of the remaining cortex shows the opposite effect (Fig. 1b, e), possibly reflecting a diminished abundance of dendritic arborisations related to thalamocortical connections, as compared to its primary sensory counterparts, hence the increasing myelin beneath the cortex yields relatively increasing contrast in association regions during the normal adolescent development. As a result, normal development features positive-signed importance for age prediction in most of the association cortex^10^, whereas this phenomenon appears generally inverse in ASD, likely reflecting reduced long-range connectivity and myelination (cf. Figure 1c, f). Supporting this notion, a recent study concluded that myelination appears a more plausible explanation of primary visual cortex thinning in childhood than pruning^42^.

The relative contrast decrease pattern appears widespread, covering most of the non-primary cortex, which might reflect long-range structural^43,44^ and functional^23,45^ cortico-cortical underconnectivity observed in ASD, whereas subcortico-cortical intrinsic functional connectivity commonly appears to be increased in ASD^46^. This notion was confirmed from a dynamical connectivity perspective^47^, and elaborated for thalamus and its connectivity with cortex^48,49^. Such an increase, however, should be interpreted with caution, considering that the thalamus has multiple nuclei heterogeneous in their intrinsic and extrinsic connectivity, yielding both decreases and increases in connectivity with cortex in ASD^50^.

With regard to diagnostic prediction, there has recently been substantial progress in development of data-driven machine learning methods using structural and functional MRI^51,52^. In our study, we aimed at diagnostic prediction using structural MRI, understanding the mechanisms of cortical contrast change, their comparison across longitudinal and cross-sectional domains, and incorporating the knowledge about these mechanisms into the model, instead of taking a fully data-driven prediction approach. The angle value distributions, for both TD and ASD populations, were well approximated by a family of extreme value distributions with virtually equal modes (Supplementary Figure 2). While this does not necessarily mean that the modes of these distributions were non-informative in estimating posterior probabilities, it does indicate a large inter-subject variability in both diagnostic groups and a possibility that a large number, if not a majority, of subjects might contribute only weakly to the overall predictive power. Some previous studies have reported a high heterogeneity across ASD/TD samples that results in no significant diagnostic group differences^18^. On the other hand, longitudinal samples generally provide less heterogeneity in diagnosis-related patterns^15^, which might explain why our diagnostic prediction model resulted in high accuracy (76% for the whole cortex and 86% for the motor system only), despite a relatively small number of subjects in the longitudinal sample.

Even though most developmental WGC change difference patterns captured by PLS analysis were bilateral, there were certain effects of lateralisation in the diagnostic prediction results. Most notably, specificity of the per-vertex models was substantially higher for the left than for the right hemisphere (Fig. 4d). A recent study on the ABIDE-I dataset observed similar left-sided laterality for the cortical thickness differences between ASD and TD subjects, as well as lateralisation of cortical pattern related to symptom severity^2^. Other researchers observed related functional MRI activation differences in the ABIDE-I sample^53,54^, followed by a growing body of research highlighting the importance of exploring functional and physiological changes in a developmental context as well^55,56^. Another recent study found disrupted diffusion connectivity patterns in the left superior longitudinal fasciculus in ASD^7^. Such lateralisation might result from a more rapid development of many cortical regions within the left hemisphere than of their right hemisphere homologues^57^, specifically language related areas, such as Broca’s, Wernicke’s and multiple regions adjacent to the arcuate fasciculus. In line with that, another diffusion MRI study identified substantial age-related change in fractional anisotropy and mean diffusivity in the superior longitudinal fasciculus in adolescence, an effect that appeared disrupted in an ASD population^8^.

### Limitations

When dealing with heterogeneous agglomerative datasets, such as ABIDE, multiple confounders may exist. Indeed, site differences constitute a major problem which cannot be fully resolved even with larger samples^18^. The approach we took allowed us to compute the metric of interest within each site, which would not be fully feasible using standard general linear model approaches, where controlling for site as a categorical variable might retain confounding effects^58^.

A second concern related to the neuropsychiatric datasets is a potential contribution of head motion to diagnostic results. We performed an assessment of motion effects on diagnostic discrimination by evaluating the gradients within the white-matter core (Supplementary Figure 4). As motion produces ‘ringing’-like artefacts on MRI images (see Supplementary Figure 9 for some examples), the variance of the gradient in a motion-contaminated image would be higher than its counterpart in an image featuring less motion. Thus, standard deviation of the gradient within the white-matter mask core can serve as a motion proxy metric (Supplementary Figure 5); it yielded no difference between ASD and TD groups (*p* = 0.3, two-sample *t*-test), and no correlation with ADOS scores (*p* = 0.1, permutation test). This approach is summarised in more detail in Supplementary Text 1. In addition to the fact that motion did not appear as a confounder in our study, it should be mentioned that previous studies^2,15^ reported increases of cortical thickness in multiple regions in ASD subjects, whereas it is known that motion causes an apparent decrease in cortical thickness^59^.

A third problem inherent in the ABIDE dataset is the small sample size and rather compromised quality of the currently available longitudinal sample. Indeed, a change in a single subject’s diagnostic label would result in 11% sensitivity change in the available longitudinal sample used in this study; this issue can only be resolved with the availability of larger samples. Regarding the data quality, we compared our quality control (QC) outcomes to the signal-to-noise ratio (SNR) values provided by MRIQC^60^ within ABIDE-II^24^. The images accepted by our QC (Supplementary Figure 6) had significantly higher SNR than those rejected (*p* < 0.01, *t*-test, see Supplementary Figure 7).

A fourth potential confounder is an inclusion of females in the longitudinal sample, whereas cross-sectional sample included only males. However, a study which also utilised the ABIDE dataset found no sex differences in cortical thickness between ASD and TD subjects, albeit such differences were found for cortical gyrification^61^. Moreover, another study designed specifically to identify possible gender predispositions in ASD, found cortical thickness in certain regions to be related to greater risk of ASD in males^62^; however, these regional differences were almost exclusively located in inferior temporal regions (cf. Figure 4). Despite these counter-examples, it is known that sex differences are related to ASD traits^63^, and thus further investigations on larger and more balanced samples must be done to understand whether and how WGC change is related to sex differences in ASD.

Another possible source of heterogeneity in the agglomerative datasets is concerned with the variability of medication taken by the subjects. ABIDE-II provides information on medication taken by a subset of subjects (most of which are ASD), which allowed us to perform preliminary qualitative evaluations regarding possible links between medication and WGC angle values (Supplementary Figure 8). Besides a few cases highlighted in that figure, it is of note that the only TD subject in the sample which was reported to have medical treatment, was incorrectly predicted as ASD. Obviously, this is a single case from a small sample, hence further investigations should be done when larger samples with more detail on medication, nutrition and other phenotypic data become more available^25,26^.

## Conclusion

We have presented an MRI study involving longitudinal and cross-sectional samples from ASD patients and age-matched TD controls. We have devised a metric, wherein the rates of change in cortical contrast are measured as differences in the contrast, relative to age. In both longitudinal and cross-sectional samples, we observed a general decrease across most of the cortex (81% cortical overlap between longitudinal and cross-sectional counterparts, see Fig. 2). The ASD subjects’ ADOS scores, as well as the severity values derived therefrom, correlated well with the cortical pattern related to ASD/TD group differences. We have implemented a Bayesian model to predict diagnostic outcomes in the longitudinal sample from its cross-sectional counterpart. Full-cortex prediction yielded 76% accuracy, constituting 83% sensitivity (ASD predicted as ASD) and 67% specificity (TD predicted as TD). Prediction based solely on bilateral motor regions achieved 83% sensitivity and 89% specificity, yielding 86% overall accuracy. The second model’s outcome suggested that the relation between BrSc values from PLS analysis and ADOS severity in the cross-sectional sample is predictive of the ADOS raw diagnostic scores in the longitudinal sample.

In the future, we plan to adapt the presented approach to other cohorts. In particular, we plan to investigate whether the explored effects take place in other age groups, especially among infants. We hypothesise that certain model adaptations might be needed, as WGC change data are likely far from being ergodic, i.e. consistent across the space-time continuum, making the longitudinal/cross-sectional inferences particularly challenging^64^. Nevertheless, with growing amounts of longitudinal and cross-sectional data available^15,25,26,65^, and with necessary model generalisations, this endeavour becomes increasingly more realistic.

## Electronic supplementary material

Supplementary Material Captions

Supplementary Table 1

Supplementary Text 1

Supplementary Text 2

Supplementary Figure 1

Supplementary Figure 2

Supplementary Figure 3

Supplementary Figure 4

Supplementary Figure 5

Supplementary Figure 6

Supplementary Figure 7

Supplementary Figure 8

Supplementary Figure 9
