# Supplementary material for: Developmental changes of cortical white–gray contrast as predictors of autism diagnosis and severity
Source: Transl Psychiatry. 2018 Nov 16;8:249. doi: 10.1038/s41398-018-0296-2 (PMC6240045; doi:10.1038/s41398-018-0296-2)
Supplement: Supplementary file 1 — Supplementary Material Captions [file 41398_2018_296_MOESM1_ESM.docx]

**SUPPLEMENTARY FIGURE AND TABLE CAPTIONS**

**Supplementary Figure 1**. A schematic depiction of the overall study concept and results. Top left inset shows how WGC is calculated: the T1 intensity values are sampled from inward and outward from the white matter surface, and the ratio between the former and the latter is obtained. Most of the cortex (shown on the brain surface) featured greater WGC decrease with age across the ASD sample (top right), and occasional greater decrease with age across the TD sample (bottom right). This brain pattern, in a multivariate context, correlated with the ASD severity measures, represented by both raw ADOS scores and proxy calibrated severity scores (bottom left).

**Supplementary Figure 2.** Specification of the cross-sectional/longitudinal predictive model. (A) Distributions of ASD and TD angles data in the cross-sectional sample are mostly different in the tails, either left or right, thus better captured by the extreme value distribution family, used as a likelihood function (C). Acquired knowledge about diagnosis-related differences from PLS is embedded into the prior function implemented as a sigmoid curve (D). Using Bayes' theorem, the posterior probability is obtained, allowing for diagnostic group prediction (B); this represents the sought-for value, i.e. the probability of a diagnostic outcome given the change of WGC with age.

**Supplementary Figure 3**. Conjunction of sensitivity and specificity of cross-sectional/longitudinal predictions based on individual vertices. The values are displayed in a conservative way such that they show either specificity or sensitivity value for a given vertex, whichever is minimal (cf. Figure 4 of the main text).

**Supplementary Figure 4**. Eroded white matter mask (WM core), shown in red, on a subject's T1 image. Standard deviation of gradient values within this eroded WM mask is computed, serving as a proxy for head motion. Orientations shown are: (a) sagittal; (b) coronal; (c) axial.

**Supplementary Figure 5**. Example T1 images illustrating image-based proxies for motion, based on the white matter (WM) gradient. Standard deviation of gradient values within eroded WM mask is lower for images with less motion (A; image ID Trinity+0050266), and higher for images with more motion (B; image ID ABIDEII-UCLA_1+29755). Of note, both images shown are from TD subjects.

**Supplementary Figure 6**. Quality control results for the longitudinal sample. Each single coronal slice represents a part of the accepted T1 image per subject per time point (baseline, top, and follow-up, bottom), with the white surface contour overlaid, shown in blue.

**Supplementary Figure 7**. Comparison of signal-to-noise ratio (SNR), as obtained from ABIDEII MRIQC for the longitudinal images that failed and passed the quality control (QC) in the current study. The images that passed the QC have significantly higher SNR than those that failed the QC (p<0.01, two-sample t-test).

**Supplementary Figure 8**. Medication taken and its association with the angle values in the cross-sectional sample. In the ASD group, atomoxetine is associated with the lower angle values, cetirizine is among the highest; of note, in the longitudinal sample (not shown here), atomoxetine taking patient (ID 51294, baseline) is correctly predicted as ASD, whereas cetirizine taking patient (ID 51234, follow-up) is incorrectly predicted as TD.

**Supplementary Figure 9**. Several examples of MRI artefacts in the UM_1 sample. (A) images contaminated by severe motion artefacts. (B) images contaminated by other artefacts, not necessarily excluding motion; top: spurious vertical and horizontal lines; middle: high-intensity blood vessels in the proximity of white matter; bottom: low SNR arising from other possible artefacts. Subject IDs are displayed.

**SUPPLEMENTARY TABLE CAPTION**

**Supplementary Table 1**. Statistics of the longitudinal sample. The columns designate, left to right: data collection site, subject ID as in NITRC database, age at baseline visit, age at follow-up visit, mean $\alpha^{lngt}$ value across cortex, raw total ADOS score, diagnostic group, diagnosis prediction using the whole-cortex model as specified in Eq.3.
