# Supplementary material for: Developmental changes of cortical white–gray contrast as predictors of autism diagnosis and severity
Source: Transl Psychiatry. 2018 Nov 16;8:249. doi: 10.1038/s41398-018-0296-2 (PMC6240045; doi:10.1038/s41398-018-0296-2)
Supplement: Supplementary file 2 — Supplementary Table 1 [file 41398_2018_296_MOESM2_ESM.docx]

| **site** | **NITRC ID** | **age, baseline** | **age, follow-up** | **angle value** | **ADOS score** | **Dx group** | **predicted as** |
| --- | --- | --- | --- | --- | --- | --- | --- |
| UCLA | 51231 | 10.28 | 12.67 | -0.04 | 14 | ASD | ASD |
| UCLA | 51234 | 10.91 | 13.5 | -0.0191 | 14 | ASD | TD |
| UCLA | 51236 | 12.42 | 15.5 | -0.0123 | 10 | ASD | ASD |
| UCLA | 51294 | 11.7 | 15.08 | -0.0127 | 9 | ASD | ASD |
| UCLA | 51298 | 10.57 | 12.58 | 0.0068 | 15 | ASD | TD |
| UCLA | 51300 | 14.08 | 17.17 | -0.0128 | 13 | ASD | ASD |
| UPSM | 50002 | 16.77 | 18.49 | -0.0073 | 12 | ASD | ASD |
| UPSM | 50006 | 13.37 | 15.11 | -0.0068 | 12 | ASD | ASD |
| UPSM | 50013 | 9.33 | 10.81 | -0.0051 | 12 | ASD | ASD |
| UPSM | 50027 | 12.24 | 13.82 | 0.0019 | 11 | ASD | ASD |
| UPSM | 50028 | 12.74 | 14.64 | -0.005 | n/a | ASD | ASD |
| UPSM | 50029 | 11.4 | 13.44 | -0.0068 | 13 | ASD | ASD |
| UCLA | 51258 | 11.55 | 14.92 | 0.0122 | - | TD | TD |
| UCLA | 51307 | 12.15 | 15.17 | -0.0078 | - | TD | TD |
| UCLA | 51313 | 13.36 | 16.16 | -0.0012 | - | TD | ASD |
| UCLA | 51315 | 13.63 | 16.76 | -0.0077 | - | TD | TD |
| UPSM | 50038 | 13.95 | 15.98 | -0.0037 | - | TD | ASD |
| UPSM | 50047 | 15.35 | 16.97 | -0.012 | - | TD | TD |
| UPSM | 50048 | 11.81 | 13.96 | -0.0005 | - | TD | ASD |
| UPSM | 50049 | 15.82 | 17.51 | 0.0134 | - | TD | TD |
| UPSM | 50050 | 14.37 | 16.1 | -0.01 | - | TD | TD |
