# Supplementary figures and images for: Developmental changes of cortical white–gray contrast as predictors of autism diagnosis and severity
Source: Transl Psychiatry. 2018 Nov 16;8:249. doi: 10.1038/s41398-018-0296-2 (PMC6240045; doi:10.1038/s41398-018-0296-2)

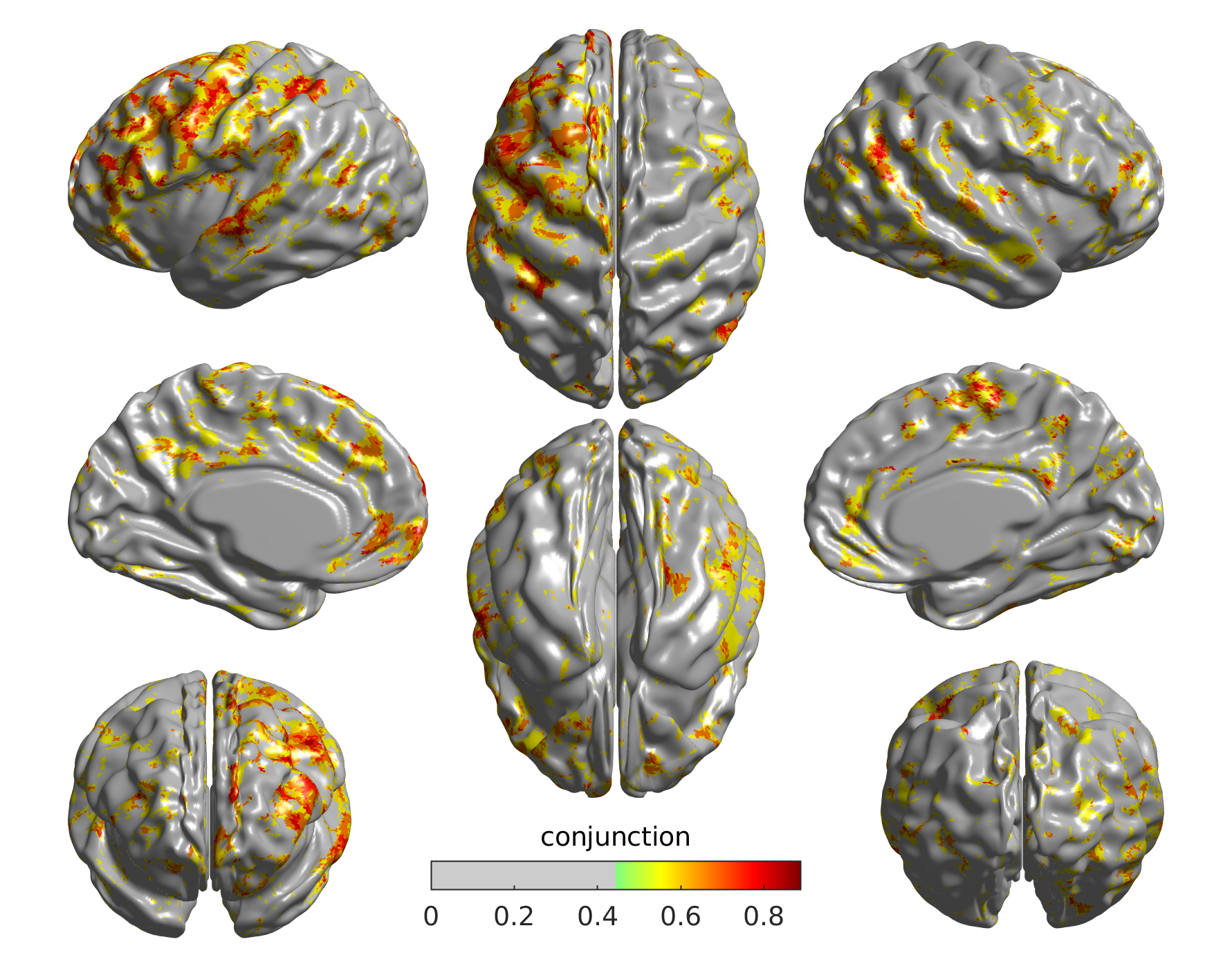

Supplement: Supplementary file 7 — Supplementary Figure 3 [file 41398_2018_296_MOESM7_ESM.tif]

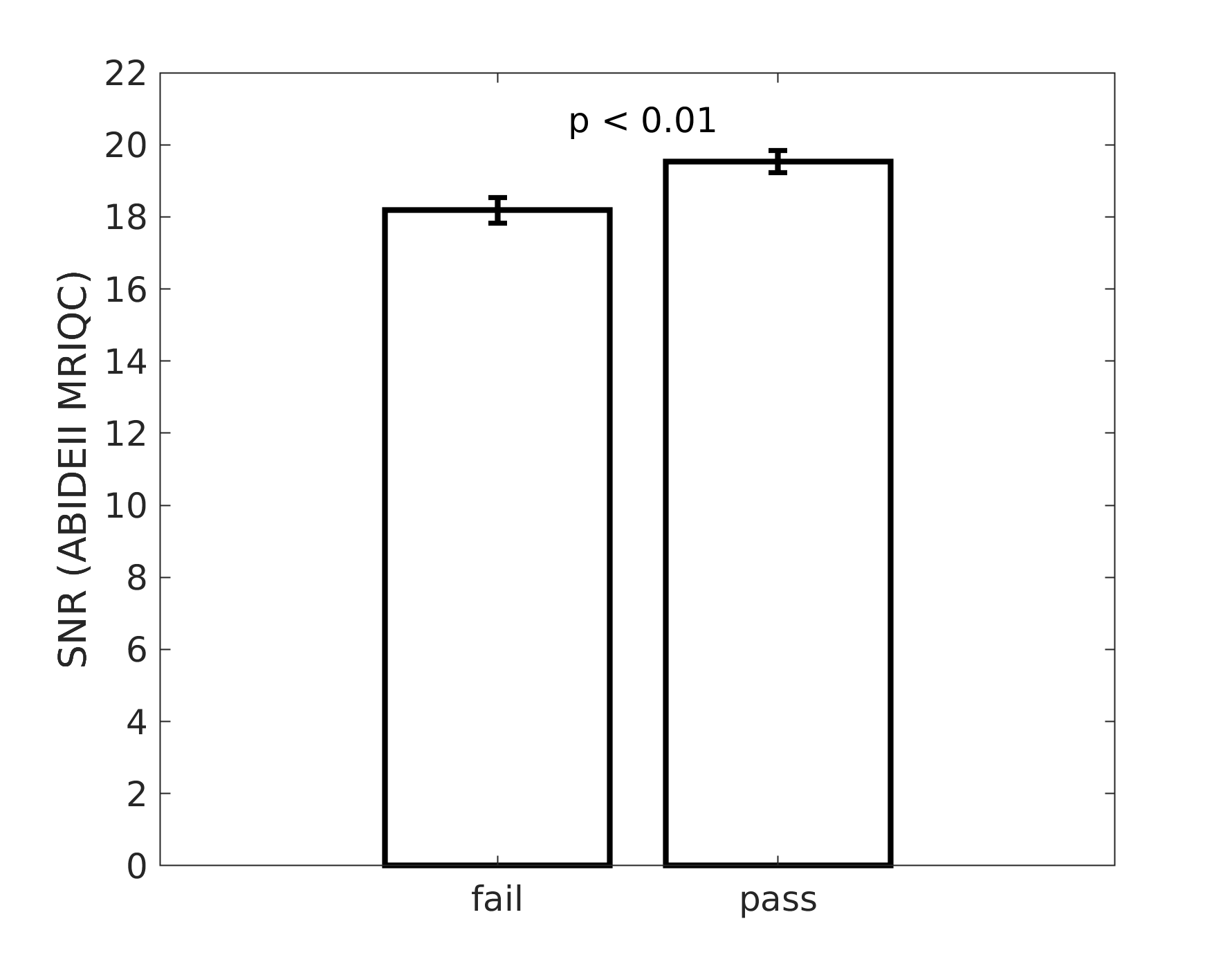

Supplement: Supplementary file 11 — Supplementary Figure 7 [file 41398_2018_296_MOESM11_ESM.tif]
